# Evaluating Cardiotoxicity in Breast Cancer Patients Treated with HER2 Inhibitors: Could a Combination of Radionuclide Ventriculography and Cardiac Biomarkers Predict the Cardiac Impact?

**DOI:** 10.3390/cancers15010207

**Published:** 2022-12-29

**Authors:** Mirela Gherghe, Alexandra Maria Lazar, Mario-Demian Mutuleanu, Cristian Ioan Bordea, Sinziana Ionescu, Raluca Ioana Mihaila, Cristina Petroiu, Adina Elena Stanciu

**Affiliations:** 1 Nuclear Medicine Department, University of Medicine and Pharmacy “Carol Davila” Bucharest, 050474 Bucharest, Romania; 2 Nuclear Medicine Department, Institute of Oncology “Professor Doctor Alexandru Trestioreanu”, 022328 Bucharest, Romania; 3 Surgical Oncology Department, University of Medicine and Pharmacy “Carol Davila” Bucharest, 050474 Bucharest, Romania; 4 Surgical Oncology Department, Institute of Oncology “Professor Doctor Alexandru Trestioreanu”, 022328 Bucharest, Romania; 5 General Surgery Department, University of Medicine and Pharmacy “Carol Davila” Bucharest, 050474 Bucharest, Romania; 6 Oncology Department, Institute of Oncology “Professor Doctor Alexandru Trestioreanu”, 022328 Bucharest, Romania; 7 Carcinogenesis and Molecular Biology Department, Institute of Oncology “Professor Doctor Alexandru Trestioreanu”, 022328 Bucharest, Romania

**Keywords:** anti-HER2 therapies, cardiotoxicity, trastuzumab, pertuzumab, cardiac biomarkers, GDF-15, ST2/IL33R, radionuclide ventriculography

## Abstract

**Simple Summary:**

Breast cancer is the most frequently diagnosed cancer in women, causing over 500,000 deaths worldwide. Human epidermal growth factor receptor 2 is overexpressed in roughly 15–20% of the breast cancer cases diagnosed nowadays, denoting poor prognoses. However, the presence of this receptor in tumoral cells provided patients with the opportunity for treatment with HER2 inhibitors, which have well-documented clinical benefits but still present variable grades of cardiotoxicity. We evaluated 22 patients, diagnosed with HER2-positive breast cancer, who underwent combination treatment with trastuzumab, pertuzumab and docetaxel, and we monitored their cardiac profiles through radionuclide ventriculography and cardiac-biomarker measurements both at the beginning of their treatment and after 6 months of therapy. Our results confirmed the good cardiac safety profile of this therapeutic scheme while also suggesting use of radionuclide ventriculography and dosing of NT-proBNP and ST2-IL33R for early detection of cardiac impairment.

**Abstract:**

(1) Background: The aim of our study was to determine whether monitoring cardiac function through RNV and cardiac biomarkers could predict the cardiac impact of combined therapy with trastuzumab, pertuzumab and docetaxel, which are regularly used nowadays to treat HER2-positive breast cancer. (2) Methods: This prospective monocentric study included 22 patients, diagnosed with HER2-positive breast cancer, who had their LVEFs and cardiac biomarkers evaluated both at the beginning of their treatment and after 6 months. Among all of the enrolled patients, two blood specimens were collected to assess circulating cardiac biomarkers. RNV was performed in each patient after “in vivo” radiolabeling of the erythrocytes. The obtained results were then statistically correlated. (3) Results: The average LVEF decrease between the two time points was approximately 4%. Of the five biomarkers we considered in this paper, only NT-proBNP correlated with the LVEF values obtained both in the baseline study and after 6 months of follow-up (r = −0.615 for T0 and r = −0.751 for T1, respectively). ST2/IL-33R proved statistically significant at the T1 time point (r = −0.547). (4) Conclusions: A combination of LVEF, NT-proBNP and ST2/IL-33R assessment may be useful for early detection of cardiac impairment in breast cancer patients treated with trastuzumab, pertuzumab and docetaxel.

## 1. Introduction

Breast cancer is the most frequently diagnosed cancer and the leading cause of cancer-related death among women, with an estimated incidence of 11.7%, representing as many as 2.2 million cases and more than 500,000 deaths attributed to it worldwide [1,2]. As this is a major public health and economic issue, research of new therapies, as well as monitoring of their safety usage, represent the main priority at present, as many studies have shown that breast cancer can be considered a curable disease. The use of a multidisciplinary approach that combines local (surgery, radiotherapy) and systemic treatment (chemotherapy, hormonal and biological therapy) has conducted to a significant reduction in mortality [3,4].

Human epidermal growth factor receptor 2 (HER2) is overexpressed in roughly 15–20% of the breast cancers diagnosed nowadays; its presence is associated with poor prognosis [1,5,6,7,8,9,10]. HER2 is a transmembrane receptor that promotes cell proliferation and diminishes apoptosis, and therefore needs tight regulation in order to prevent abnormal cell growth from occurring. The main HER2 inhibitors used to treat HER2-positive metastatic breast cancer are the monoclonal antibodies trastuzumab and pertuzumab, which are mainly associated with anthracyclines, but other therapeutic schemes can include taxanes, vinorelbine, platinum salts, hormonal therapy (anastrozole) or other HER2 inhibitors (lapatinib and ado-trastuzumab) [8,11,12,13]. The clinical benefits of HER2 inhibitors are well-documented. However, the important cardiac adverse reactions must be taken into consideration.

Cardiotoxicity is a type of cardiomyopathy, usually caused by substances (medications, especially cancer treatments, or intrinsic toxins) that damage either the structure or the electrophysiology of the heart muscle. After secondary malignancies, cardiovascular disease is the leading cause of morbidity and mortality among breast cancer survivors [14,15]. Trastuzumab-induced cardiotoxicity exhibits differently from anthracycline-induced cardiomyopathy [11,16]. Trastuzumab is directly cardiotoxic or may potentiate the cardiotoxic effect of anthracyclines due to an effect on myocardial ErbB2 tyrosine-protein kinase receptors, which have a protective role on the cardiac function [17]. In this case, the risk of cardiac illness is not related to the cumulative dose, granting the possibility of a partial reversibility of the cardiac decline, usually just through ceasing administration of the drug. This sustains the concept that HER2 inhibition is of functional and not structural consequence [16,18].

Assessment of left ventricular (LV) function and its volumes is particularly important in patients treated with HER2 inhibitors, since it provides valuable information about the prognosis and follow-up of therapeutic protocol. Both echocardiography and planar equilibrium radionuclide ventriculography (RNV) have been used for a long time, but for the past few years, single-photon emission computed tomography (SPECT) and cardiac magnetic resonance (CMR) have gained a lot of popularity for their ability to estimate left ventricular ejection fractions (LVEFs) more accurately. While CMR is still largely unavailable because of its technical restrictions and cost issues, RNV is widely used, with little disadvantages [5,19,20].

In addition to LVEF monitoring, both the European Society of Cardiology (ESC) and the American Heart Association (AHA), as well as the European Society for Medical Oncology (ESMO), in their guidelines regarding cardiotoxicity of different cancer treatments, recommend the measurement of circulating cardiac biomarkers, such as cardiac troponin I (cTn I) and the amino-terminal fragment of the prohormone brain-type natriuretic peptide (NT-proBNP), to evaluate the cardiac dysfunction induced via HER2-targeted therapies [18,21,22]. From the natriuretic peptide category, the amino-terminal fragment of the prohormone atrial-type natriuretic peptide (NT-proANP) could have comparable diagnostic and prognostic accuracy, but this is less-documented. Other emerging biomarkers that may predict cardiac involvement and the risk of heart failure (HF), such as interleukin-33R (IL-33R), which is also known as suppression of tumorigenicity 2 protein/interleukin-33R (ST2/IL-33R) and growth differentiation factor 15 (GDF-15), have been recently studied [23,24]. While ST2/IL-33R is used as an additional stratification factor for HF, revealing cardiac stress and fibrosis [25,26], GDF-15 plays a double role: a cardioprotective role in HF, reducing recruitment of polymorphonuclear cells that contribute to cardiac tissue destruction, and a function as a diagnostic and prognostic factor in cancer development and progression [27].

In this study, we assessed cardiac function using planar RNV and SPECT, according to the definition of cardiotoxicity related to cancer therapeutics stated by the ESC as a decrease in LVEF over 10%-points to below 50% [18]. These results correlated with the values obtained via measuring each patient’s circulating cardiac biomarkers (NT-proBNP, NT-proANP, cTn I, GDF-15 and ST2/IL-33R).

## 2. Materials and Methods

### 2.1. Patient Selection

Our study included prospective data from 51 female patients enrolled after full understanding and agreeing to the institutional informed consent. This study was conducted in the Nuclear Medicine Department of the Oncology Institute of Bucharest “Prof. Dr. Alexandru Trestioreanu”, Romania, from September 2020 to March 2022. The reference population was represented by female patients previously diagnosed with breast tumors prior to beginning treatment with HER2 inhibitors. The eligibility criteria included (1) presenting breast tumors, with at least an equivocal HER2 status (2+) on immunohistochemical examinations, that were later confirmed through a fluorescence “in situ” hybridization (FISH) test; (2) being recommended a treatment protocol that included both trastuzumab and pertuzumab, along with docetaxel; and (3) having a baseline LVEF, estimated with echocardiography, of over 50%. Patients were questioned about their personal and family medical histories and were each required to provide their last echocardiographic examination for inclusion in this study. Each patient had two blood samples drawn for cardiac biomarker assessment before the “in vivo” labeling of the erythrocytes.

Between the baseline and the follow-up RNV scans, patients underwent a treatment regimen that included an initial administration of 8 mg/kg of trastuzumab, 840 mg of pertuzumab and 75 mg/m^2^ of docetaxel, followed by a regimen compounded of 6 mg/kg of trastuzumab, 420 mg of pertuzumab and 100 mg/m^2^ of docetaxel, administered through intravenous infusion every 21 days, for at least seven (±one) cycles.

**Figure 1:**
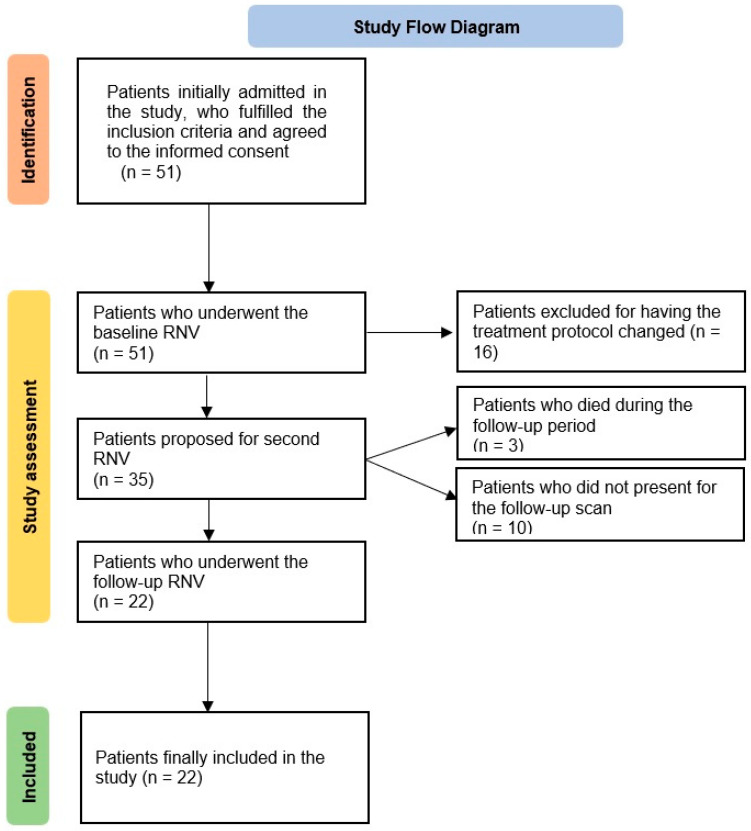
Flow diagram illustrating the patient-selection process. Abbreviations: RNV—radionuclide ventriculography.

Among the 51 patients who were first selected, 16 patients, who presented metastatic disease and initially received trastuzumab, pertuzumab and docetaxel as adjuvant therapies, were excluded due to a change in treatment protocol. Three other patients died during the follow-up period of this study. Additionally, 10 patients who initially agreed to participate in this study were not present for the follow-up scan; thus, 22 patients were included in our final research (Figure 1).

### 2.2. Erythrocyte Labeling

Labeling of erythrocytes was performed according to the Society of Nuclear Medicine and Molecular Imaging (SNMMI)–European Association of Nuclear Medicine (EANM) joint guideline for gated equilibrium radionuclide angiography [28], using the “in vivo” technique, that involves two intravenous injections: one containing stannous pyrophosphate and one containing ^99m^Tc-pertechnetate. Patients were initially administered 1 mL of stannous pyrophosphate (Technescan^™^ PYP), dissolved in a sterile isotonic NaCl solution, 20 min before the injection of a dose of 555–740 MBq (15–20 mCi) of sodium pertechnetate ^99m^Tc.

A reduction of ^99m^Tc-pertechnetate is needed in order to facilitate binding to hemoglobin; this is achieved through administration of stannous ions (Sn^2+^) as stannous pyrophosphate, with free Sn^2+^ ions passively diffusing into the cell. Next, ^99m^Tc-pertechnetate, administered in the second injection, crosses the red-cell membrane via diffusion or the band-3 anion transport system, being intracellularly reduced by the Sn^2+^ ions. The reduced ^99m^Tc-pertechnetate then attaches to the β-chain of the hemoglobin [28].

Although believed to have a lower labeling efficiency, this method results in satisfying diagnostic quality, eliminating the risk and the requirements of “in vitro” labeling and handling of human blood while being more cost-effective [28].

### 2.3. Imaging Acquisition Technique and Reconstruction

Imaging acquisition protocol was carried out in accordance to the SNMMI/EANM joint guideline for gated equilibrium radionuclide angiography [28]. The examination protocol included planar RNV and blood-pool single photon emission computed tomography/computed tomography (SPECT/CT) scans, performed for 10 min, following administration of sodium pertechnetate ^99m^Tc. Images were acquired using a Discovery 670 DR SPECT/CT system (General Electric Healthcare, Chicago, IL, USA) equipped with low-energy, high-resolution (LEHR) collimators. A planar RNV scan was carried out with the detectors in the L position, according to the following parameters: left anterior oblique position at 45 degrees for detector 1, a maximum acquisition time of 600 s, a 140 keV ± 10% energy window, 16 frames/cycle, a matrix size of 64 × 64 and a zoom factor of 2.2×. Electrocardiogram triggering had a 30% beat rejection window (±15%).

Blood-pool SPECT (BP-SPECT) of the thoracic region was acquired using the detectors in the same position, a dual energy window of 140 keV ± 10% and 120 ± 5%, a matrix size of 64 × 64, a zoom of 2.2×, 16 frames/cycle, a step-and-shoot rotation time of 40 s/view, a 90° arc per detector, and a view angle of 3°, for a total of 60 views. Gated RNV triggering had a 30% beat rejection window (±15%). The SPECT acquisition was completed with a low-dose CT scan for scatter correction, performed maintaining the position of the patient, with a 5 mm slice thickness, at 120 kV, with 20 mA and dose modulation enabled (GE smart scan).

Two experienced physicians performed the postprocessing of the acquired images and interpreted the results.

Planar images were processed using an ejection fraction analysis application (GE Xeleris 4.0, General Electric Healthcare, Chicago, IL, USA), using the automatic mode, adjusting the limiting and the background regions of interest (ROIs) when necessary. The parameters that described left ventricular ejection fractions were obtained from the phase–activity curve in concordance with the corresponding phase histogram.

SPECT-CT images were reconstructed using QBS software (Version 2009, Cedars-Sinai Medical Center, CA, USA), after manual positioning of the ventricular ROI, using filtered back projection (FBF) reconstruction, Butterworth filtering and MDC motion correction (General Electric Healthcare), with deactivated scatter correction. Images were generated based on the parametric amplitude, and LVEFs were calculated using count-based volumes, with a left ventricular threshold of 35%.

### 2.4. Biomarker Assessment

In all enrolled patients, two blood specimens were collected via venipuncture and placed into BD Vacutainer SSTTM II Advance tubes with clot activators (silica particles) before “in vivo” labeling of the erythrocytes. Serum samples were obtained via clotting (30 min, room temperature) and centrifugation (15 min at 1000× *g* and 4 °C). After that, they were aliquoted into labeled cryovials and frozen at −80 °C for up to 12 months.

NT-proBNP (amino acids 1-76) and NT-proANP (amino acids 1-98), secreted from the cardiac ventricles and atria, respectively, have longer half-lives, are more stable and consequently are more reliable analytes of prolonged cardiac overload than are biologically active peptides (BNP and ANP), as shown by The Study Group on Biomarkers in Cardiology (ESC Working Group on the Acute Cardiac Care) [29]. However, we investigated the stability of NT-proBNP (1-76) and NT-proANP (1-98). Storage at −80 °C for 12 months resulted in NT-proBNP and NT-proANP concentrations decreasing between 0.3% and 1.2% compared with samples thawed after 24 h of storage.

Serum concentrations of NT-proBNP (1-76), NT-proANP (1-98), cTn I, ST2/IL-33R and GDF-15 were measured using commercially available quantitative enzyme-linked immunosorbent assay kits (NT-proBNP and NT-proANP from Biomedica Medizinprodukte GmbH, Vienna, Austria; cTn I from Abcam, Cambridge, UK; Human ST2/IL-33R and GDF-15 Quantikine ELISA kits from R&D Systems, Inc., Minneapolis, MN, USA). The serum samples required a 20-fold dilution for ST2/IL-33R and a 4-fold dilution for GDF-15 in different diluents. According to the manufacturer, the values of the intra-assay precision were similar to those of the inter-assay precision, with coefficients of variation ranging from 6.0 to 8.0% for NT-proBNP; 2.0 to 5.1% for NT-proANP; 3.0 to 7.1% for cTn 1; 4.4 to 5.6% for ST2/IL-33R and 1.8 to 5.6% for GDF-15. However, precision (intra-assay variation) was tested with 8 measurements of 3 different samples of known concentrations in 1 assay, and the reproducibility (interassay variation) for the same 3 samples was tested 8 times in 2 assays. The intra- and interassay CVs were as follows: 3.1% and 3.9%, respectively, at a mean concentration of 563.7 pmol/L for NT-proBNP; 2.3% and 3.2%, respectively, at a mean concentration of 1.07 nmol/L for NT-proANP; 4.5% and 5.6%, respectively, at a mean concentration of 10.27 ng/mL for ST2/IL-33R; 2.8% and 3.6%, respectively, at a mean concentration of 889.3 ng/mL for GDF-15.

The lower limits of detection were 3 pmol/L for NT-proBNP, 0.05 nmol/L for NT-proANP, 2.45 pg/mL for ST2/IL-33R and 2.0 pg/mL for GDF-15.

All assays were performed in duplicate according to the manufacturer’s recommendations and in a way that minimized any effects of repeated freeze–thaw cycles.

### 2.5. Statistical Analysis

Data analysis was performed using IBM SPSS Statistics Version 26 (IBM, SPSS, Inc., Chicago, IL, USA, 2019). The results were presented as mean ± standard deviation. A paired samples t-test was used to compare the results obtained in the two phases of this study, with a *p*-value < 0.05 considered to be statistically significant. Correlations between the LVEF values and the studied biomarkers were made using a Pearson correlation test. Scatter plots were drawn to illustrate the correlations between the different parameters.

## 3. Results

All patients’ descriptive characteristics are presented in Table 1. The mean age in our study group was 55.55 ± 9.89, in a range from 38 to 74 years old. Only 40.9% of patients presented cardiac risk factors at enrollment in our study, the most consistent associations being dyslipidemia (27.27%), essential arterial hypertension (18.18%) and diabetes mellitus (13.63%). Dyslipidemia was assessed as circulating total cholesterol ≥4mmol/L (≥155 mg/dL) or triglycerides ≥ 1.7 mmol/L (≥150 mg/dL), as stated by the ESC guidelines [30].

Out of the 22 patients included in our study, 19 (86.36%) had followed previous treatment protocols that included potentially cardiotoxic drugs, such as association of an anthracycline and an alkylating agent (usually epirubicin and cyclophosphamide—40.9% cases) or taxanes (docetaxel/paclitaxel—77.3% cases), as shown in Table 1. Seven patients (31.81%) have had both types of the aforementioned chemotherapeutic protocols in their previous treatment regimens.

A percentage of 59.1% of patients (n = 13) received the therapeutic scheme as a neoadjuvant treatment before lumpectomy or mastectomy, while the rest, 40.9% (n = 9), underwent the protocol as adjuvant therapy after the primary treatment. None of our patients, neither those with neoadjuvant therapy nor those treated adjuvantly, received radiotherapy, targeted on the heart area during the follow-up period, that might have had an impact on cardiac function.

We defined the baseline investigations as T0 and the follow-up examinations performed after 6 months of treatment as T1. The LVEFs and circulating biomarkers measured in the blood samples collected at these two time points are shown in Table 2. All of the results are expressed as mean and standard deviation. In consideration of the analyzed biomarkers, a statistically significant difference was observed in the case of NT-proBNP (*p* = 0.013), presenting an average increase of 2.2-fold (between T0 and T1). The values of the other biomarkers changed between the two time points (27.5% and 73.3% increases in NT-proANP and cTn I, respectively; 5.6% and 18.8% decreases in ST2/IL-33R and GDF-15, respectively), but without any statistical significance.

**Figure 2:**
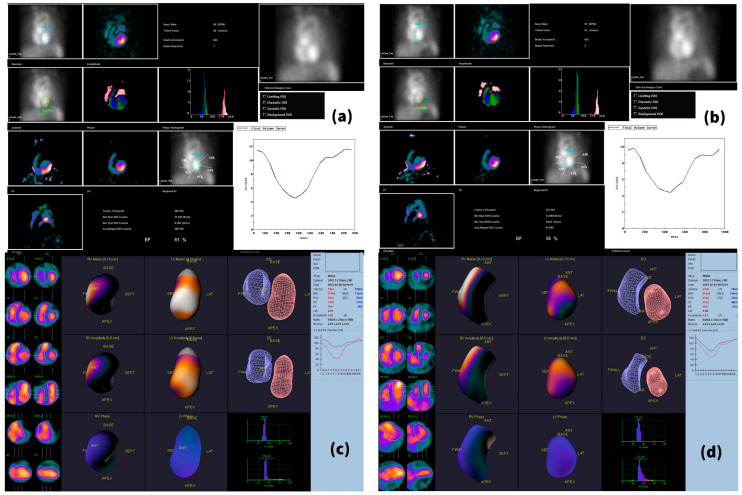
Patient presenting a small decrease in LVEF, not fulfilling the ESC criteria for cardiotoxicity. This 55-year-old female patient suffered from HER2 2+ invasive lobular carcinoma (confirmed through FISH), ER- and PR-negative, previously treated with taxanes. During the 6-month period, she received eight cycles of trastuzumab, pertuzumab and docetaxel. A small decline in LVEF can be observed in both planar and SPECT images, with the EF diminishing from 61% at T0 (**a**) to 56% at T1 (**b**) on the planar scans, and from 64% (T0—(**c**)) to 59% (T1—(**d**)) on the SPECT acquisitions.

**Figure 3:**
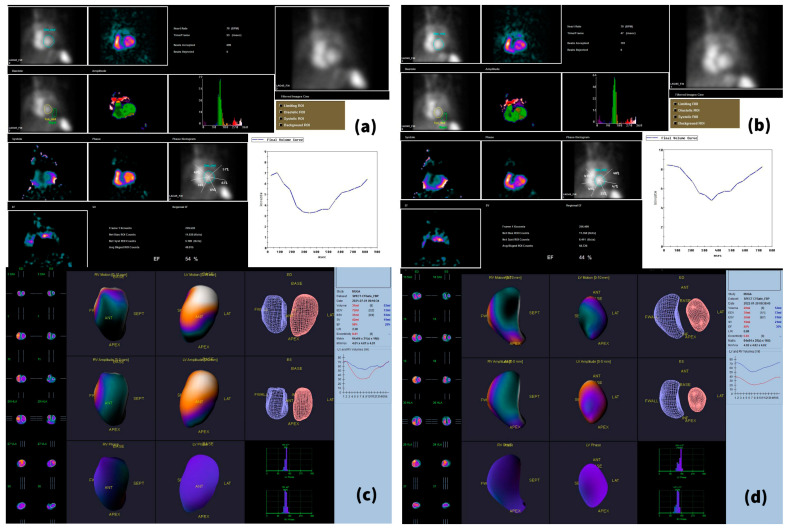
Patient fulfilling the ESC criteria for cardiotoxicity. This 64-year-old female patient suffered from HER2 3+ invasive ductal carcinoma, ER- and PR-negative, previously treated with anthracyclines. During the 6-month period, she received six cycles of trastuzumab, pertuzumab and docetaxel. A decline in LVEF can be observed in both planar and SPECT images, with the EF diminishing from 54% at T0 (**a**) to 44% at T1 (**b**) on planar scans, and from 58% (T0—(**c**)) to 48% (T1—(**d**)) on the SPECT acquisitions.

The baseline LVEF was 57.77 ± 6.21%. After 6 months of combined treatment with trastuzumab, pertuzumab and docetaxel, 19 patients showed decreased LVEF, resulting in a mean LVEF at T1 of 53.36 ± 7.30%, with an average decrease of approximately 4% (Table 2 and Figure 2). When comparing the LVEFs obtained in the planar scan with those that resulted from the BP-SPECT, we observed that the ejection fractions obtained in the SPECT examination were slightly higher than those obtained in gated planar images; LVEF means ± SD of 57.95 ± 5.89% for T0 BP-SPECT and of 54.95 ± 6.89% for T1 BP-SPECT were obtained (Table 2). Five patients (22.72%) fulfilled the ESC criteria for cardiotoxicity, presenting a decrease in LVEF of over 10%, to below 50%, between the baseline study and T1 (Figure 3).

For correlation purposes, we used only the LVEFs obtained on the planar images, as this used to be considered the “gold standard” in LVEF calculations. The correlation coefficients between the LVEF values and the measured biomarkers are illustrated in Table 3.

**Figure 4:**
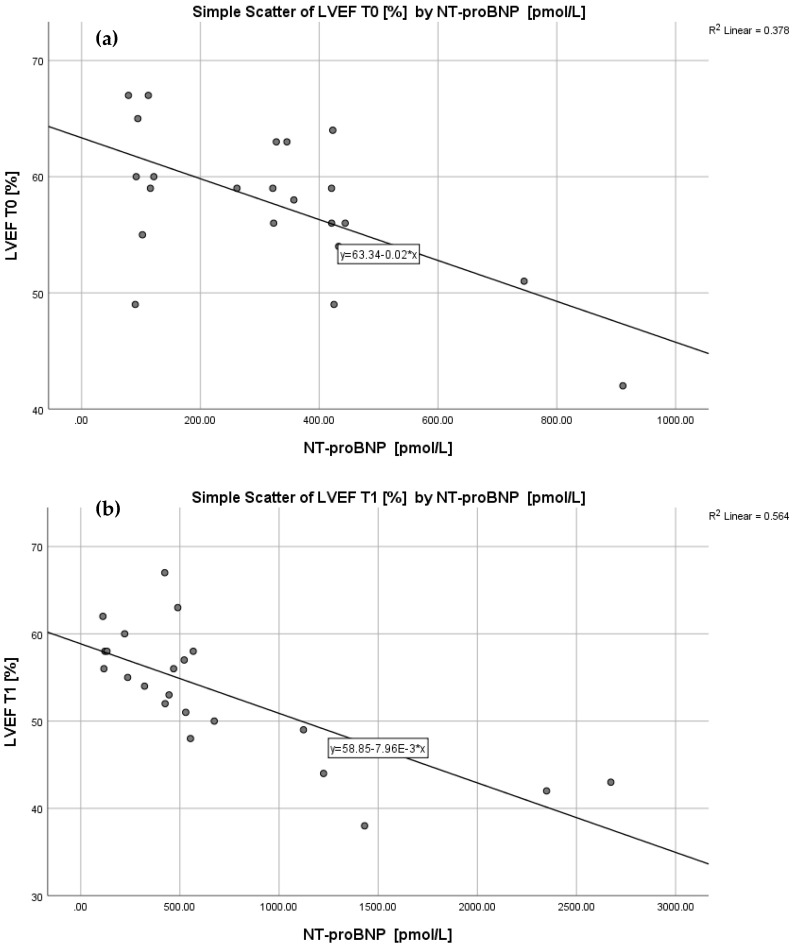
Correlation between NT-proBNP and LVEFs at T0 (**a**) and T1 (**b**). Abbreviations: NT-proBNP—amino-terminal fragment of the prohormone brain-type natriuretic peptide; LVEF—left ventricular ejection fraction; T0—baseline scan; T1—follow-up scan.

Of the five biomarkers we considered in this paper, only NT-proBNP correlated with the LVEF values obtained both in the baseline study and after 6 months of follow-up (r = −0.615, *p* = 0.002 for T0 and r = −0.751, *p* < 0.001 for T1, respectively). As can be seen from Figure 4a,b, the correlation was negative: as the NT-proBNP increases, the LVEFs decreases, confirming the increased myocardial strain in these patients. Another parameter that proved statistically significant at the T1 time point was ST2/IL-33R (r = −0.547, *p* = 0.008), indicating the existence of cardiac stress in the patients following treatment with trastuzumab and pertuzumab. The values of the other cardiac biomarkers evaluated in our study, although varying between the two examinations, proved no statistically relevant correlation with the LVEFs obtained at the two time points.

## 4. Discussion

Our study investigated whether LVEF assessment through RNV, which is known to provide more accurate measurements than echocardiography, and dosing of specific circulating cardiac biomarkers could evaluate cardiac damage inflicted by combined therapy with trastuzumab, pertuzumab and docetaxel in patients suffering from breast cancer.

Numerous studies have demonstrated the superiority of combined therapy with trastuzumab and pertuzumab, together with adjuvant chemotherapy, in treating both metastatic and nonmetastatic breast cancer [31,32,33,34,35]. While the cardiotoxic risks presented by both pertuzumab and trastuzumab have been widely researched [5,6,31], a clear outcome of the degree of myocardial damage and its reversibility has not been reached yet. Furthermore, a consensus on standard cardiac monitoring and an explicit definition of the cardiotoxicity induced with chemotherapy need to be developed to better manage cancer patients.

Several studies researched the cardiotoxic potential of the therapeutic scheme administered to our patients: trastuzumab and pertuzumab, together with docetaxel. A paper published by Gianni et al. [32] regarding the NeoSphere trial reported an occurrence of cardiac adverse reactions in three out of one hundred and seven patients from a group that underwent therapy with trastuzumab, pertuzumab and docetaxel; these patients showed an LVEF decline of 10–15% from baseline, to less than 50%, during the neoadjuvant period, but with LVEF improvement to more than 50% by cycle 4.

The CLEOPATRA clinical trial [33] outlined a higher incidence of any grade of left ventricular systolic dysfunction in the control group (that received no pertuzumab) than in the study group (8.3% vs. 4.4%), with an LVEF decrease of more than 10 points from the baseline in 6.6% cases of the control group vs. 3.8% in the group that additionally received pertuzumab, concluding that the combination of trastuzumab with pertuzumab and docetaxel did not increase the risk of symptomatic or asymptomatic cardiac dysfunction. A later paper by Swain et al. [34], published in 2015 as a follow-up of that study, stated that LVEF reduction of more than 10% from the baseline to an absolute value lower than 50%, found initially in 24 out of 394 patients, was reversed in 21 patients.

Another study that researched the efficacy of pertuzumab, trastuzumab and anthracycline- and taxane-based neoadjuvant chemotherapy was the BERENICE trial [35], which reported an incidence of 2% (four patients) LVEF decline in the group that was treated with docetaxel, pertuzumab and trastuzumab after previously receiving four cycles of fluorouracil, epirubicin and cyclophosphamide (FEC). Declines in the neoadjuvant period were generally reversible, often with recovery by the time of the next LVEF assessment, and no patient in this group experienced any HF event.

The TRYPHAENA trial [36], which analyzed combinations of trastuzumab and pertuzumab with various other chemotherapeutic protocols, outlined for Arm B of their study population—which received FEC for three cycles, followed by docetaxel, trastuzumab and pertuzumab for cycles four through six—incidences of symptomatic LVEF decrease of 5.3% during neoadjuvant treatment and of 12.3% during adjuvant therapy, all of these being improved to over 50% at the data cutoff. In a later follow-up of this trial, Schneeweiss et al. [37] demonstrated that this combination is generally well tolerated, with no additional safety signals, and reported that during the entire study period, only two patients (2.75%) from the group that was administered docetaxel had symptomatic cardiac events.

All of the studies mentioned above revealed a great cardiac safety profile for this combined therapeutic protocol, with generally low occurrences of symptomatic LVEF decline, in contrast to the incidence of 22.75% found in our study population. However, in consideration that our patient lot was small (twenty-two individuals) and pondering the number of patients that fulfilled the ESC criteria in the aforementioned studies (up to eight patients), the five patients affected by symptomatic LVEF decline fit the number interval related to previous studies. Furthermore, the evaluation method needs to be considered when these results are analyzed. We used equilibrium radionuclide ventriculography as the main tool to evaluate the baseline and follow-up LVEFs, achieving uniformity throughout this study, while the trials discussed above monitored cardiac function through either echocardiography or RNV.

While monitoring cardiac function through imaging methods remains mandatory for HER2-positive breast cancer patients, cardiac damage without reflection in function changes should also be pondered; therefore, cardiac biomarkers should be carefully assessed. Only specific cardiac biomarkers can detect subtle and early changes in cardiac function (myocardial injury, dysfunction, inflammation and fibrosis), as they may precede and predict the development of left ventricular impairment. A recent review published by Bouwer et al. [5] suggests considering the measurements of troponin, NT-proBNP, C-reactive protein, myeloperoxidase, immunoglobulin E and ST2 when observing patients on treatment protocols that include trastuzumab. One study, conducted by Goel et al. [38], analyzed the potential predictive values of cTn I and NT-proBNP in evaluation of cardiotoxicity in female patients with normal cardiac function and who received trastuzumab for breast cancer; the authors reported elevated NT-proBNP levels in a substantial proportion of patients with normal LVEFs, but no significant changes in cTn I levels, suggesting that this might reflect the mechanism of cardiotoxicity as a subclinical cardiac strain. Another paper, by Sawaya et al. [39], further expanded that research, studying, in addition to cTnI and NT-proBNP, the potential benefit of recording levels of ST2 as predictors of cardiotoxicity for therapeutic protocols that include trastuzumab. In contrast to Goel et al., this study revealed no notable changes in the levels of NT-proBNP and ST2 but found that elevated cTn I concentrations (>30 pg/mL) were predictive of subsequent cardiotoxicity. The 2017 American College of Cardiology/American Heart Association Guideline for the Management of Heart Failure [26] recommends measuring levels of cTn I, NT-proBNP and ST2/IL-33 when anticipating cardiac involvement and possible HF.

Until the present day, no paper has been published on the utility of monitoring cardiac biomarkers in patients following treatment with both pertuzumab and trastuzumab, together or not with other chemotherapeutic medication, with our study being the first to research their potential use in this particular case. Therefore, we investigated and evaluated the dynamics of multiple circulating biomarkers related to combined treatment with trastuzumab, pertuzumab and docetaxel as follows: cTn I for cardiomyocyte damage; NT-proBNP for increased myocardial wall stress; NT-proANP for hemodynamic overload of atria; ST2/IL-33R for myocardial inflammation, myocyte stress/stretch, myocardial interstitial fibrosis and remodeling of myocardial collagen fibers; and GDF-15 for its cardioprotective role. NT-proANP is released in response to cardiac wall stretch from the atrial tissue. The lack of correlation between NT-proANP and LVEF in our patients indicates that the increased NT-proANP secretion after combined treatment had no extension from the atria to the ventricles. NT-proANP can show acute volume overload and hemodynamic changes, while NT-proBNP better reflects prolonged overload. Our results agree with those obtained by Goel et al. for trastuzumab-based therapy [38]. Statistically significant negative correlation, measured in our study, between serum NT-proBNP concentrations and LVEFs (r = −0.751, *p* < 0.001) reinforces the idea that the mechanism of cardiotoxicity in the case of HER2 inhibitors is due to increased myocardial strain and not to cardiomyocyte injury, as would be suggested from the increases of 73.3% in cTn I level [5]. The nonsignificant statistical association between cTn I and LVEF (r = −0.315, *p* = 0.153) underlies this statement. The ST2/IL-33R pathway is expressed in the failing human heart, and its expression is associated with myocardial inflammation, stress and profibrotic signaling proteins. Although ST2/IL-33R slightly decreased, the statistically significant correlation between the levels of ST2/IL-33R and the LVEF values at T1 (r = −0.547, *p* = 0.008) indicates the existence of cardiovascular stress after 6 months of treatment with trastuzumab, pertuzumab and docetaxel.

A recent paper, published by Kirkham et al. [40], evaluated the role GDF-15 and other circulating biomarkers might play in analyzing cardiac involvement in patients treated with trastuzumab, observing that levels of GDF-15 raised between baseline and cycle four of treatment but reverted to values close to those of baseline on cycle seventeen, suggesting that trastuzumab initially induces transitory myocardial inflammation and oedema that rapidly resolves without any changes in cardiac function. A review by May et al. [41] states that GDF-15 proved to be an important predictor of cardiac adverse reactions and mortality, independent of ejection fraction and serum levels of NT-proBNP. However, it is known that GDF-15 also functions as a tumoral marker in cases of breast cancer being linked to occurrences of metastasis and trastuzumab resistance [42,43].

In our study population, the mean levels of GDF-15 declined from the baseline values after 6 months of treatment with trastuzumab, pertuzumab and docetaxel, but no statistically significant correlation between these values and the resulting LVEFs was found. The 18.8% decrease in serum GDF-15 concentration might have come as a response to the therapeutic scheme rather than to changes in cardiac function, suggesting that, in our lot of individuals, GDF-15 might have functioned as a tumoral marker and not as a predictor of HF.

Our findings might raise the potential of better investigating novel HER2-targeted therapies for breast cancer, such as trastuzumab deruxtecan and trastuzumab emtansine, that present greater efficacy than the current treatment regimens present [44]. Recent studies have shown that neither of the two treatment options possesses cardiotoxic risk [45,46,47]; however, most of the studies assessed LVEF only with echocardiography, without considering the additional information brought by cardiac biomarkers, thus making this a great opportunity for research in this field.

There are, however, some limitations to our study that need to be considered. Firstly, our study population was compounded of only 22 female patients, which might affect the applicability of our results on a larger scale. Secondly, the method we used for cardiac function evaluation, equilibrium radionuclide ventriculography, is strongly operator-dependent, and although two physicians analyzed the obtained images, these results might present a grade of variability. Thirdly, the cardiac affliction given from the previous treatments undergone by our patients could have played a role in the results, given the inability to accurately appreciate the cardiac damage inflicted on a molecular level by previous treatment with anthracyclines and taxanes.

## 5. Conclusions

A combination assessment of LVEF and circulating biomarkers such as NT-proBNP and ST2/IL-33R may become useful for early detection of cardiac impairment in monitoring of breast cancer patients treated with trastuzumab, pertuzumab and docetaxel, enabling physicians to change or completely stop treatment when needed. Nevertheless, further extensive studies, on larger patient populations, need to be performed, with the aim of applying this algorithm in clinical practice.

## Figures and Tables

**Table 1:** Patient characteristics. Abbreviations: PR—progesterone receptor; ER—estrogen receptor, HER2—human epidermal growth factor receptor 2.

Patient Characteristic	Variable	Value
Age (Mean ± Standard Deviation)		55.55 ± 9.89
Tumor Grading (%)	G2	45.5%
	G3	54.5%
PR (%)	Positive	59.1%
	Negative	40.9%
ER (%)	Positive	54.5%
	Negative	45.5%
HER2 Status	2+	40.9%
	3+	50.1%
Cardiac Risk Factors (%)	Yes	40.9%
	No	59.1%
Metastatic Disease	Yes	54.5%
	No	45.5%
Previous Chemotherapy	Anthracyclines	40.9%
	Taxanes	77.3%

**Table 2:** The values of planar and BP-SPECT LVEFs and circulating biomarkers for each time point. Abbreviations: LVEF—left ventricular ejection fraction; NT-proBNP—amino-terminal fragment of the prohormone brain-type natriuretic peptide; NT-proANP—amino-terminal fragment of the prohormone atrial-type natriuretic peptide; cTnI—cardiac troponin I; sT2/IL-33R—suppression of tumorigenicity 2 protein/interleukin-33R; GDF-15—growth differentiation factor 15. All data are expressed as mean ± standard deviation.

Variable	T0	T1	*p*-Value
Planar LVEF (%)	57.77 ± 6.21	53.36 ± 7.30	0.003
BP-SPECT LVEF (%)	57.95 ± 5.89	54.95 ± 6.89	0.035
NT-proBNP (pmol/L)	316.69 ± 217.18	689.14 ± 688.24	0.013
NT-proANP (nmol/L)	0.69 ± 1.01	0.88 ± 1.23	0.339
cTnI (pg/mL)	7.51 ± 15.28	13.17 ± 29.37	0.141
ST2/IL-33R (ng/mL)	24.20 ± 20.73	23.34 ± 26.74	0.882
GDF-15 (ng/mL)	1522.55 ± 1724.26	1236 ± 1132.94	0.513

**Table 3:** Correlation coefficients between the LVEFs and the circulating biomarkers measured for the T0 and T1 time points. Abbreviations: r—correlation coefficient; cTnI—cardiac troponin I; LVEF—left ventricular ejection fraction; NT-proBNP—amino-terminal fragment of the prohormone brain-type natriuretic peptide; NT-proANP—amino-terminal fragment of the prohormone atrial-type natriuretic peptide; sT2/IL-33R—suppression of tumorigenicity 2 protein/interleukin-33R; GDF-15—growth differentiation factor 15.

Biomarker	*r* Planar LVEF T0	*p*	*r* SPECT LVEF T0	*p*	*r* Planar LVEF T1	*p*	*r SPECT* LVEF T1	*p*
NT-proBNP	−0.615	0.002	−0.679	0.001	−0.751	0.000	−0.785	0.000
NT-proANP	0.280	0.207	0.175	0.436	0.417	0.053	0.258	0.246
cTnI	0.046	0.838	0.114	0.614	−0.315	0.153	−0.372	0.088
ST2/IL-33R	0.016	0.942	−0.092	0.684	−0.547	0.008	−0.514	0.014
GDF-15	0.251	0.260	0.220	0.325	−0.191	0.396	−0.224	0.316

## Data Availability

All the data generated or analyzed during this study are included in the manuscript.

## References

[1] Ishii K., Morii N., Yamashiro H. (2019). Pertuzumab in the Treatment of HER2-Positive Breast Cancer: An Evidence-Based Review of Its Safety, Efficacy, and Place in Therapy. Core Evid..

[2] Gherghe M., Mutuleanu M.-D., Stanciu A.E., Irimescu I., Lazar A., Bacinschi X., Anghel R.M. (2022). Quantitative Analysis of SPECT-CT Data in Metastatic Breast Cancer Patients—The Clinical Significance. Cancers.

[3] Ben-Dror J., Shalamov M., Sonnenblick A. (2022). The History of Early Breast Cancer Treatment. Genes.

[4] Gherghe M., Bordea C., Blidaru A. (2015). Clinical Significance of the Lymphoscintigraphy in the Evaluation of Non-Axillary Sentinel Lymph Node Localization in Breast Cancer. Chirurgia.

[5] Bouwer N.I., Jager A., Liesting C., Kofflard M.J.M., Brugts J.J., Kitzen J.J.E.M., Boersma E., Levin M.-D. (2020). Cardiac Monitoring in HER2-Positive Patients on Trastuzumab Treatment: A Review and Implications for Clinical Practice. Breast.

[6] Alhussein M.M., Mokbel A., Cosman T., Aghel N., Yang E.H., Mukherjee S.D., Dent S., Ellis P.M., Dhesy-Thind S., Leong D.P. (2021). Pertuzumab Cardiotoxicity in Patients With HER2-Positive Cancer: A Systematic Review and Meta-Analysis. CJC Open.

[7] Jerusalem G., Lancellotti P., Kim S.-B. (2019). HER2+ Breast Cancer Treatment and Cardiotoxicity: Monitoring and Management. Breast Cancer Res. Treat..

[8] Agunbiade T.A., Zaghlol R.Y., Barac A. (2019). Heart Failure in Relation to Tumor-Targeted Therapies and Immunotherapies. Methodist Debakey Cardiovasc. J..

[9] Mohan N., Jiang J., Dokmanovic M., Wu W.J. (2018). Trastuzumab-Mediated Cardiotoxicity: Current Understanding, Challenges, and Frontiers. Antib. Ther..

[10] Hussain Y., Drill E., Dang C.T., Liu J.E., Steingart R.M., Yu A.F. (2019). Cardiac Outcomes of Trastuzumab Therapy in Patients with HER2-Positive Breast Cancer and Reduced Left Ventricular Ejection Fraction. Breast Cancer Res. Treat..

[11] Nemeth B.T., Varga Z.V., Wu W.J., Pacher P. (2017). Trastuzumab Cardiotoxicity: From Clinical Trials to Experimental Studies. Br. J. Pharmacol..

[12] Tan-Chiu E., Yothers G., Romond E., Geyer C.E., Ewer M., Keefe D., Shannon R.P., Swain S.M., Brown A., Fehrenbacher L. (2005). Assessment of Cardiac Dysfunction in a Randomized Trial Comparing Doxorubicin and Cyclophosphamide Followed by Paclitaxel, With or Without Trastuzumab As Adjuvant Therapy in Node-Positive, Human Epidermal Growth Factor Receptor 2–Overexpressing Breast Cancer: NSABP B-31. JCO.

[13] Dang C.T., Yu A.F., Jones L.W., Liu J., Steingart R.M., Argolo D.F., Norton L., Hudis C.A. (2016). Cardiac Surveillance Guidelines for Trastuzumab-Containing Therapy in Early-Stage Breast Cancer: Getting to the Heart of the Matter. JCO.

[14] Haque R., Prout M., Geiger A.M., Kamineni A., Thwin S.S., Avila C., Silliman R.A., Quinn V., Yood M.U. (2014). Comorbidities and Cardiovascular Disease Risk in Older Breast Cancer Survivors. Am. J. Manag. Care.

[15] Henry M.L., Niu J., Zhang N., Giordano S.H., Chavez-MacGregor M. (2018). Cardiotoxicity and Cardiac Monitoring among Chemotherapy-Treated Breast Cancer Patients. JACC Cardiovasc. Imaging.

[16] Nowsheen S., Aziz K., Park J.Y., Lerman A., Villarraga H.R., Ruddy K.J., Herrmann J. (2018). Trastuzumab in Female Breast Cancer Patients With Reduced Left Ventricular Ejection Fraction. J. Am. Heart Assoc..

[17] Florescu M., Cinteza M., Vinereanu D. (2013). Chemotherapy-Induced Cardiotoxicity. Maedica.

[18] Zamorano J.L., Lancellotti P., Rodriguez Muñoz D., Aboyans V., Asteggiano R., Galderisi M., Habib G., Lenihan D.J., Lip G.Y.H., Lyon A.R. (2016). 2016 ESC Position Paper on Cancer Treatments and Cardiovascular Toxicity Developed under the Auspices of the ESC Committee for Practice Guidelines: The Task Force for Cancer Treatments and Cardiovascular Toxicity of the European Society of Cardiology (ESC). Eur. Heart J..

[19] Ribeiro M.L., Jorge A.J.L., Nacif M.S., Martins W.d.A. (2019). Early Detection and Monitoring of Cancer Chemotherapy-Related Left Ventricular Dysfunction by Imaging Methods. Arq. Bras. Cardiol..

[20] Sachpekidis C., Sachpekidis V., Moralidis E., Arsos G. (2018). Equilibrium Radionuclide Ventriculography: Still a Clinically Useful Method for the Assessment of Cardiac Function?. Hell. J. Nucl. Med..

[21] Alexandre J., Cautela J., Ederhy S., Damaj G.L., Salem J., Barlesi F., Farnault L., Charbonnier A., Mirabel M., Champiat S. (2020). Cardiovascular Toxicity Related to Cancer Treatment: A Pragmatic Approach to the American and European Cardio-Oncology Guidelines. J. Am. Heart Assoc..

[22] Curigliano G., Lenihan D., Fradley M., Ganatra S., Barac A., Blaes A., Herrmann J., Porter C., Lyon A.R., Lancellotti P. (2020). Management of Cardiac Disease in Cancer Patients throughout Oncological Treatment: ESMO Consensus Recommendations. Ann. Oncol..

[23] Dudek M., Kałużna-Oleksy M., Migaj J., Straburzyńska-Migaj E. (2020). Clinical Value of Soluble ST2 in Cardiology. Adv. Clin. Exp. Med..

[24] Rochette L., Dogon G., Zeller M., Cottin Y., Vergely C. (2021). GDF15 and Cardiac Cells: Current Concepts and New Insights. Int. J. Mol. Sci..

[25] Vianello E., Dozio E., Tacchini L., Frati L., Corsi Romanelli M.M. (2019). ST2/IL-33 Signaling in Cardiac Fibrosis. Int. J. Biochem. Cell Biol..

[26] Yancy C.W., Jessup M., Bozkurt B., Butler J., Casey D.E., Colvin M.M., Drazner M.H., Filippatos G.S., Fonarow G.C., Givertz M.M. (2017). 2017 ACC/AHA/HFSA Focused Update of the 2013 ACCF/AHA Guideline for the Management of Heart Failure: A Report of the American College of Cardiology/American Heart Association Task Force on Clinical Practice Guidelines and the Heart Failure Society of America. Circulation.

[27] Assadi A., Zahabi A., Hart R.A. (2020). GDF15, an Update of the Physiological and Pathological Roles It Plays: A Review. Pflug. Arch. Eur. J. Physiol..

[28] Farrell M.B., Galt J.R., Georgoulias P., Malhotra S., Pagnanelli R., Rischpler C., Savir-Baruch B. (2020). SNMMI Procedure Standard/EANM Guideline for Gated Equilibrium Radionuclide Angiography*. J. Nucl. Med. Technol..

[29] Thygesen K., Mair J., Mueller C., Huber K., Weber M., Plebani M., Hasin Y., Biasucci L.M., Giannitsis E., Lindahl B. (2012). Recommendations for the Use of Natriuretic Peptides in Acute Cardiac Care†: A Position Statement from the Study Group on Biomarkers in Cardiology of the ESC Working Group on Acute Cardiac Care. Eur. Heart J..

[30] Mach F., Baigent C., Catapano A.L., Koskinas K.C., Casula M., Badimon L., Chapman M.J., De Backer G.G., Delgado V., Ference B.A. (2020). 2019 ESC/EAS Guidelines for the Management of Dyslipidaemias: Lipid Modification to Reduce Cardiovascular Risk: The Task Force for the Management of Dyslipidaemias of the European Society of Cardiology (ESC) and European Atherosclerosis Society (EAS). Eur. Heart J..

[31] Sanctis R.D., Giordano L., D’Antonio F., Agostinetto E., Marinello A., Guiducci D., Masci G., Losurdo A., Zuradelli M., Torrisi R. (2021). Clinical Predictors of Cardiac Toxicity in HER2-Positive Early Breast Cancer Patients Treated with Adjuvant s.c. versus i.v. Trastuzumab. Breast.

[32] Gianni L., Pienkowski T., Im Y.-H., Roman L., Tseng L.-M., Liu M.-C., Lluch A., Staroslawska E., de la Haba-Rodriguez J., Im S.-A. (2012). Efficacy and Safety of Neoadjuvant Pertuzumab and Trastuzumab in Women with Locally Advanced, Inflammatory, or Early HER2-Positive Breast Cancer (NeoSphere): A Randomised Multicentre, Open-Label, Phase 2 Trial. Lancet Oncol..

[33] Baselga J., Cortés J., Kim S.-B., Im S.-A., Hegg R., Im Y.-H., Roman L., Pedrini J.L., Pienkowski T., Knott A. (2012). Pertuzumab plus Trastuzumab plus Docetaxel for Metastatic Breast Cancer. N. Engl. J. Med..

[34] Swain S.M., Baselga J., Kim S.-B., Ro J., Semiglazov V., Campone M., Ciruelos E., Ferrero J.-M., Schneeweiss A., Heeson S. (2015). Pertuzumab, Trastuzumab, and Docetaxel in HER2-Positive Metastatic Breast Cancer. N. Engl. J. Med..

[35] Swain S.M., Ewer M.S., Viale G., Delaloge S., Ferrero J.-M., Verrill M., Colomer R., Vieira C., Werner T.L., Douthwaite H. (2018). Pertuzumab, Trastuzumab, and Standard Anthracycline- and Taxane-Based Chemotherapy for the Neoadjuvant Treatment of Patients with HER2-Positive Localized Breast Cancer (BERENICE): A Phase II, Open-Label, Multicenter, Multinational Cardiac Safety Study. Ann. Oncol..

[36] Schneeweiss A., Chia S., Hickish T., Harvey V., Eniu A., Hegg R., Tausch C., Seo J.H., Tsai Y.-F., Ratnayake J. (2013). Pertuzumab plus Trastuzumab in Combination with Standard Neoadjuvant Anthracycline-Containing and Anthracycline-Free Chemotherapy Regimens in Patients with HER2-Positive Early Breast Cancer: A Randomized Phase II Cardiac Safety Study (TRYPHAENA). Ann. Oncol..

[37] Schneeweiss A., Chia S., Hickish T., Harvey V., Eniu A., Waldron-Lynch M., Eng-Wong J., Kirk S., Cortés J. (2018). Long-Term Efficacy Analysis of the Randomised, Phase II TRYPHAENA Cardiac Safety Study: Evaluating Pertuzumab and Trastuzumab plus Standard Neoadjuvant Anthracycline-Containing and Anthracycline-Free Chemotherapy Regimens in Patients with HER2-Positive Early Breast Cancer. Eur. J. Cancer.

[38] Goel S., Simes R.J., Beith J.M. (2011). Exploratory Analysis of Cardiac Biomarkers in Women with Normal Cardiac Function Receiving Trastuzumab for Breast Cancer. Asia-Pac. J. Clin. Oncol..

[39] Sawaya H., Sebag I.A., Plana J.C., Januzzi J.L., Ky B., Tan T.C., Cohen V., Banchs J., Carver J.R., Wiegers S.E. (2012). Assessment of Echocardiography and Biomarkers for the Extended Prediction of Cardiotoxicity in Patients Treated With Anthracyclines, Taxanes, and Trastuzumab. Circ. Cardiovasc. Imaging.

[40] Kirkham A.A., Pituskin E., Thompson R.B., Mackey J.R., Koshman S.L., Jassal D., Pitz M., Haykowsky M.J., Pagano J.J., Chow K. (2022). Cardiac and Cardiometabolic Phenotyping of Trastuzumab-Mediated Cardiotoxicity: A Secondary Analysis of the MANTICORE Trial. Eur. Heart J. Cardiovasc. Pharmacother..

[41] May B.M., Pimentel M., Zimerman L.I., Rohde L.E. (2021). GDF-15 as a Biomarker in Cardiovascular Disease. Arq. Bras. Cardiol..

[42] Wischhusen J., Melero I., Fridman W.H. (2020). Growth/Differentiation Factor-15 (GDF-15): From Biomarker to Novel Targetable Immune Checkpoint. Front. Immunol..

[43] Windrichova J., Fuchsova R., Kucera R., Topolcan O., Fiala O., Finek J., Slipkova D. (2017). MIC1/GDF15 as a Bone Metastatic Disease Biomarker. Anticancer Res..

[44] Cortés J., Kim S.-B., Chung W.-P., Im S.-A., Park Y.H., Hegg R., Kim M.H., Tseng L.-M., Petry V., Chung C.-F. (2022). Trastuzumab Deruxtecan versus Trastuzumab Emtansine for Breast Cancer. N. Engl. J. Med..

[45] Bardia A., Harnden K., Mauro L., Pennisi A., Armitage M., Soliman H. (2022). Clinical Practices and Institutional Protocols on Prophylaxis, Monitoring, and Management of Selected Adverse Events Associated with Trastuzumab Deruxtecan. Oncologist.

[46] Acibuca A., Sezer A., Yilmaz M., Sumbul A.T., Demircan S., Muderrisoglu I.H., Ozyilkan O. (2021). Cardiotoxicity of Trastuzumab Emtansine (T-DM1): A Single-Center Experience. J. Int. Med. Res..

[47] Barroso-Sousa R., Tarantino P., Tayob N., Dang C., Yardley D.A., Isakoff S.J., Valero V., Faggen M., Mulvey T., Bose R. (2022). Cardiac Outcomes of Subjects on Adjuvant Trastuzumab Emtansine vs Paclitaxel in Combination with Trastuzumab for Stage I HER2-Positive Breast Cancer (ATEMPT) Study (TBCRC033): A Randomized Controlled Trial. NPJ Breast Cancer.

